# Association between Diverticulosis and Colorectal Neoplasia: Analysis from a Large Austrian Database

**DOI:** 10.3390/jcm13206078

**Published:** 2024-10-12

**Authors:** Andreas Völkerer, Sarah Wernly, Georg Semmler, Maria Flamm, Mathias Ausserwinkler, Leonora Datz, Nikolaus Götz, Hannah Hofer, Elmar Aigner, Christian Datz, Bernhard Wernly

**Affiliations:** 1 Department of Internal Medicine, General Hospital Oberndorf, Teaching Hospital of the Paracelsus Medical University, 5020 Salzburg, Austria; a.voelkerer@kh-oberndorf.at (A.V.); sarah@wernly.net (S.W.); l.datz@kh-oberndorf.at (L.D.); hannah.hofer@bbsalz.at (H.H.); bernhard@wernly.net (B.W.); 2 Division of Gastroenterology and Hepatology, Department of Medicine III, Medical University of Vienna, 1090 Vienna, Austria; georg.semmler@meduniwien.ac.at; 3 Institute of General Practice, Family Medicine and Preventive Medicine, Paracelsus Medical University, 5020 Salzburg, Austria; maria.flamm@pmu.ac.at; 4 Department of Internal Medicine, Elisabethinen Hospital Klagenfurt, 9020 Klagenfurt, Austria; mathias.ausserwinkler@gmx.at; 5 Clinic I for Internal Medicine, University Hospital Salzburg, Paracelsus Medical University, 5020 Salzburg, Austria; e.aigner@salk.at; 6 Department of Internal Medicine, General Hospital Sankt Vinzenz, 6511 Zams, Austria; nikolaus.goetz@krankenhaus-zams.at

**Keywords:** diverticulosis, colorectal neoplasia, advanced adenoma, polyps

## Abstract

**Background:** Colorectal neoplasia and diverticulosis are common findings on colonoscopies. While adenomas are precursors to colorectal cancer, diverticulosis is usually asymptomatic but can lead to diverticulitis. Despite their prevalence and coexistence, the relationship between these conditions remains unclear. This study investigates whether diverticulosis is associated with adenomas, considering shared risk factors and potential inflammation-driven mechanisms. **Methods:** We examined 6154 asymptomatic individuals undergoing colorectal cancer screening in Austria. Diverticulosis and colorectal neoplasia were documented during screenings based on macroscopic definitions. Advanced neoplasia was defined as polyps >1 cm or high-grade dysplasia. Associations between diverticulosis and neoplastic findings were assessed using univariate and multivariable logistic regression models. **Results:** Although the overall incidence of any polypoid lesion was higher in the diverticulosis group (37% vs. 30%), statistical analysis revealed a comparable rate of advanced neoplasms in both groups. Importantly, no significant link between diverticulosis and advanced neoplasms was found (OR 1.125; 95% CI: 0.933 to 1.357, *p* = 0.218) even after adjusting for confounding factors. In a univariate analysis, a statistically significant association between diverticulosis and the presence of any colorectal polyps was identified (OR 1.388; 95% CI: 1.244–1.549, *p* < 0.0001). However, after adjusting for confounding factors in model 2 (OR 1.065, 95% CI: 0.942 to 1.204, *p* = 0.314) and model 3 (OR 1.071, 95% CI: 0.925 to 1.239, *p* = 0.360), this effect was also not statistically significant. **Conclusions:** Patients with diverticulosis share demographic and clinical features with those at risk of colorectal neoplasia, such as older age, male gender, and higher cardiometabolic risk. However, diverticulosis does not independently increase the risk of advanced colorectal neoplasia or unspecified polypoid lesions.

## 1. Introduction

The health of the gut encompasses a variety of conditions that can significantly affect an individual’s quality of life in the long term. The occurrence of adenomas and the formation of diverticula are two widespread conditions. Adenomas are benign tumors that have the potential to progress to colorectal cancer if left untreated [1]. With increasing prevalence with age and a higher occurrence in males, around 18% of patients aged 45–50 and 25% of patients aged 50–59 exhibit colonic adenomas [2]. Despite available prevention methods, colorectal cancer is considered a leading cause of cancer morbidity and mortality in developed countries [3].

Diverticulosis refers to usually asymptomatic [4,5] protrusions of the colon mucosa, which can be found in about 30% of individuals under 60 and about 70% of those over 80, [6] often as an incidental finding. Although these diverticula normally do not cause symptoms, about 10–25% of patients eventually develop symptoms. These include symptomatic uncomplicated diverticular disease (SUDD) or, in severe cases, diverticulitis (4%), perforations, and bleeding [7,8,9].

The occurrence of adenomas and diverticulosis is influenced by common risk factors such as age, male gender, metabolic components, dietary habits, and lifestyle [10,11]. Despite the frequency, the mutual relationship is obviously unclear and has been the subject of several controversial studies [12,13,14,15].

This research aims to analyze and investigate an association between diverticulosis and colorectal adenomas or cancer in an asymptomatic Central European CRC screening population, considering common risk and confounding factors as well as the possibility of an inflammation-mediated neoplastic mechanism. To investigate this scientific question, we analyze data from a single-center, epidemiological, retrospective, observational, non-interventional, and uncontrolled cohort study. The study includes participants from the ‘Salzburger Kolon-Karzinom Prävention und Intervention’ (SAKKOPI) project. This research was carried out at the General Hospital Oberndorf, located near Salzburg, Austria, with the focus on colorectal cancer screening, which is recommended in Austria beginning at the age of 45. By analyzing the prevalence, distribution, and potential interrelationship of these conditions, we seek to gain a deeper understanding of their coexistence and their impact on bowel health management.

## 2. Materials and Methods

### 2.1. Study Population

Individuals participating in the prospective registry study “Salzburg Colon Cancer Prevention Initiative” (SAKKOPI) were included in this retrospective single-center cohort study. This cohort comprises asymptomatic individuals who underwent colorectal cancer screening at the General Hospital in Oberndorf, Austria, during the period from January 2007 to March 2020. Patients from the general population were enrolled either through referral by their primary care physician or by self-enrollment in an opportunistic screening program, which was fully funded by health insurance, making it free of charge for the participants. The study comprised a total sample size of 6154 participants, all of whom underwent routine screening colonoscopies between one and three times. Each participant provided written informed consent, permitting the use of their data for scientific research purposes.

**Figure 1:**
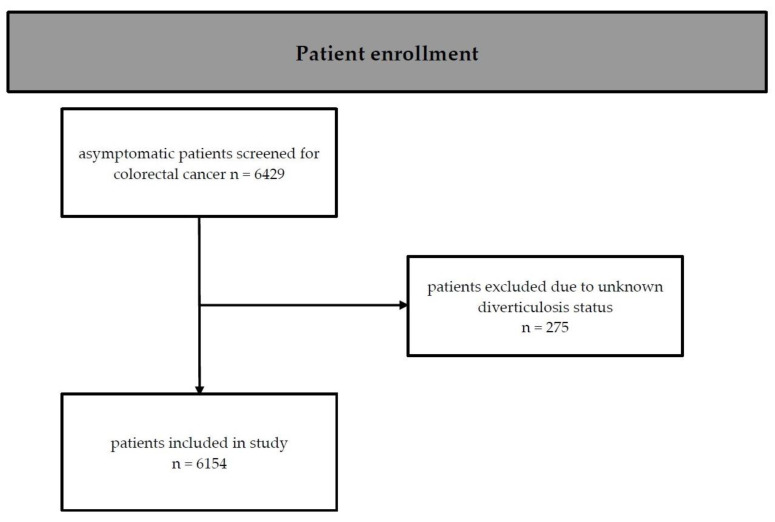
Flow diagram of patient enrollment.

Clinical and laboratory data were collected for all participants, who also completed a medical history questionnaire. We defined and calculated body mass index (BMI), arterial hypertension, smoking status, dyslipidemia, and metabolic syndrome in accordance with current guidelines [16,17,18]. All patients from the study cohort who had valid data relevant to the research question, particularly regarding the presence of colonic diverticulosis, were included in our analysis. The process of patient enrollment is illustrated in Figure 1. This cohort has already been extensively described in previous studies [19].

### 2.2. Patient Assessment for Risk Factors

All patients were examined over a period of 2 consecutive days. On the first day, upon hospital admission, their vital signs were recorded, clinical assessments were performed, and laboratory tests were conducted: To gather the necessary data, a comprehensive medical history was obtained through an interview process (clinical history). This anamnesis, along with the results of the physical examination, was recorded in the medical software (Patidok 2.0, Professional Clinical Software GmbH, Klagenfurt, Austria). Vital signs were measured using certified blood pressure monitors and pulse oximeters, with the digital data entered into the system. Electrocardiogram (ECG) results were directly imported from certified devices into the medical software for further medical evaluation. Height, weight, and measurements of waist and hip circumference were input into the system by trained nursing staff, with the BMI automatically calculated afterward. Research staff then manually extracted the required data from the digitized medical records and anonymously transferred it into a database (Microsoft Access 16.0, Microsoft, Redmond, WA, USA). Additional data were collected through a paper-based questionnaire, which included dichotomous questions, group categories, rating scales with graded responses, as well as single- and multiple-choice options with supplemental selections. The questionnaire gathered further information on diet, physical activity, and social factors. Numerical data on dietary habits and physical activity were also collected. Patients provided details about their family and medical history via the questionnaire. In instances where there were uncertainties or missing information in the questionnaire or clinical history, participants were contacted by phone or mail for clarification. Blood samples were mostly analyzed in the hospital’s internal laboratory. The laboratory tests covered routine parameters such as complete blood count, electrolytes, liver and kidney function, pancreatic enzymes, inflammation markers, lipids, blood coagulation, iron and copper metabolism, erythrocyte sedimentation rate, protein electrophoresis, and glucose metabolism markers (including insulin levels, HbA1c, and oral glucose tolerance test). Each participant underwent a standardized abdominal ultrasound and a FibroScan, which included the Controlled Attenuation Parameter (CAP) and elasticity median to assess liver fibrosis and steatosis. The second day was dedicated to performing colonoscopies. All colonoscopies were carried out by default by experienced endoscopists. Patients were sedated using Propofol or a combination of Propofol and Midazolam. Body mass index (BMI) was calculated based on World Health Organization (WHO) standards, and arterial hypertension was categorized according to the European Society of Cardiology (ESC) guidelines on blood pressure control [17]. The classification of smoking behavior was divided into “never smokers” and “active smokers” based on participant-reported information. Metabolic syndrome was defined according to the criteria set forth by the IDF/AHA/NHLBI consensus [18]. Additionally, a family history of colorectal carcinoma (CRC) was evaluated, and patients were classified as having a positive family history if they had a first-degree relative (parent, sibling, or child) diagnosed with CRC.

### 2.3. Assessment of Colorectal Lesions and Diverticulosis

The colonoscopy procedure followed international guidelines, ensuring that all established performance standards were achieved [20]. Based on the findings, patients were classified into one of the following groups: “no diverticulosis”, “left-sided diverticulosis”, “right-sided diverticulosis”, or “pandiverticulosis”. All polyps underwent histopathologic examination and were classified according to their macroscopic and microscopic characteristics. Advanced colorectal neoplasia was defined as an adenoma measuring ≥1 cm, exhibiting high-grade dysplasia or villous features, or as histologically confirmed colorectal carcinoma [21]. The category of any polypoid lesion encompasses hyperplastic polyps, adenoma, advanced neoplasms, serrated lesions, and carcinoma.

### 2.4. Statistical Analysis

Continuous data were evaluated using either the Mann–Whitney U test or the Student’s *t* test, based on the data distribution. The findings are reported as median  ±  interquartile range (IQR) or mean  ±  standard deviation (SD), respectively. Categorical data are presented as frequencies (percentages) and were analyzed with the chi-square test. All statistical tests were two-tailed, and a *p*-value of less than 0.05 was deemed significant. The primary endpoint was advanced neoplasia presence, with the secondary endpoint focusing on any polyp detection identified during screening colonoscopy. The definition of advanced colorectal neoplasia was as previously established [21]. The primary exposure was the presence or absence of diverticula, and the primary endpoint was advanced colorectal neoplasia. The secondary endpoint was any macroscopic polypoid lesion. Model 1 represents the univariate analysis. We performed a multivariable logistic regression, adjusting for age, sex, metabolic syndrome, and educational level (model 2), and additionally for alcohol consumption, red meat intake, fast food consumption, vegetable and fruit intake, and physical activity (Model 3). We further conducted sensitivity analyses for sex, age (above and below 55 years), obesity (BMI > 30), and the presence of metabolic syndrome using model 1. The odds ratios (ORs) with their 95% confidence intervals (CIs) were derived for the dichotomous outcome variables. Statistical evaluations were carried out with StataNow version 18.5 (StataCorp LLC, College Station, TX, USA).

## 3. Results

**Figure 2:**
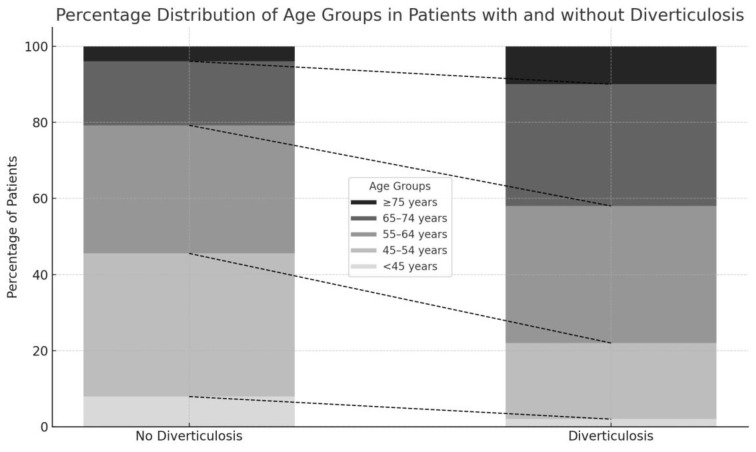
Percentage distribution of age groups among patients with and without diverticulosis.

In this study involving 6154 individuals, significant differences were observed based on the presence of diverticulosis, particularly regarding age, gender and the prevalence of metabolic syndrome. In our study population, the median age of patients without diverticulosis is 56 years (IQR: 51–63), while for those with diverticulosis, the median age is significantly higher at 62 years (IQR: 55–69 years). When further stratified by age groups, it becomes clear that younger patients are less likely to have diverticulosis. For instance, only 2% of patients with diverticulosis are under 45 years old, compared to 8% in the non-diverticulosis group. A similar pattern is observed in the 45–54 age group. The distribution becomes more balanced in the 55–64 age group. However, among patients with diverticulosis, 32% fall within the 65–74 age range, while only 17% of those without diverticulosis are in this age group. This upward trend continues in the oldest age group, highlighting a strong correlation between advancing age and the likelihood of developing diverticulosis (Figure 2).

This trend is particularly noteworthy given the parallel increase in the incidence of colorectal neoplasia with advancing age, underscoring the importance of investigating the potential independent association between diverticulosis and colorectal neoplasia.

In the group without diverticulosis (N = 3877), females accounted for 50% (1930) of the population, whereas in the diverticulosis group (N = 2277), they comprised only 44% (1009), highlighting a significant gender disparity (*p* < 0.001). This finding suggests a potential male predominance in the occurrence of diverticulosis. Moreover, metabolic syndrome was more prevalent in individuals with diverticulosis, affecting 84% (1906), versus 73% (2836) of those without (*p* < 0.001) (Table 1).

**Figure 3:**
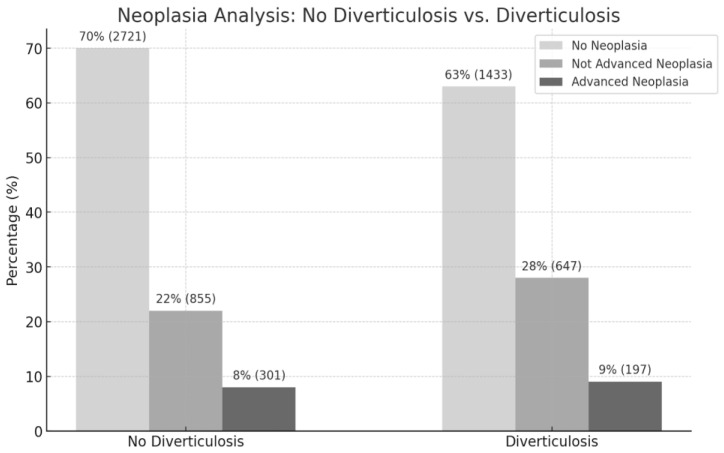
Adenoma rates in the corresponding diverticulosis category.

Overall, 9% (197 individuals) with diverticulosis and 8% (301 individuals) without diverticulosis had advanced neoplasia (Figure 3).

Specifically, the OR for advanced lesions in individuals with diverticulosis was 1.125 (95% CI: 0.933 to 1.357, *p* = 0.218), suggesting no significant link between diverticulosis and advanced neoplasias. Furthermore, the multivariable analysis in model 2 that adjusted for age, sex, metabolic syndrome, and education level (OR 0.844, 95% CI: 0.688 to 1.037, *p* = 0.106) and in model 3 that additionally adjusted for alcohol consumption, red meat intake, fast food consumption, vegetable and fruit intake, and physical activity (OR 0.797, 95% CI: 0.626 to 1.016, *p* = 0.067) reinforced the lack of a significant association. The sensitivity analysis performed also showed no evidence of an association between diverticulosis and advanced neoplasia (Table 2).

Regarding the occurrence of “any polypoid lesion”, 37% (844 individuals) of the diverticulosis group and 30% (1156 individuals) of the group without diverticulosis were affected (Figure 3). In the univariate analysis, a statistically significant association between diverticulosis and the presence of any polyp was identified, with an odds ratio of 1.388 (95% CI: 1.244 to 1.549, *p* < 0.0001), indicating that individuals with diverticulosis were more likely to have a polyp. This was also confirmed in the conducted sensitivity analysis (Table 3).

However, this association was not observed in the multivariable analysis after adjusting for confounding factors in model 2 (OR 1.065, 95% CI: 0.942 to 1.204, *p* = 0.314) and in model 3 (OR 1.071, 95% CI: 0.925 to 1.239, *p* = 0.360), suggesting that the association seen in the univariate analysis might be influenced by these confounders.

## 4. Discussion

In recent decades, several studies have examined the relationship between diverticulosis and colorectal polyps. However, these studies are very heterogeneous due to different study designs, differently defined patient groups and indications for colonoscopy, as well as various ethnicities. A review of this topic seems particularly useful in terms of the clinical relevance of a potentially more intensive early detection program when associating these two common local findings for cancer prevention.

The results of our study significantly contribute to the literature regarding the relationship between diverticulosis and colorectal neoplasms in an asymptomatic screening population. While our study showed an increased overall incidence of any polypoid lesions in the diverticulosis group, logistic regression analysis revealed that the rate of advanced neoplasms was comparable between the groups. Even though the univariate analysis showed a statistically significant association between diverticulosis and the presence of any polyps, no association could be determined after adjusting for confounding factors such as age, gender, metabolic syndrome, and education in model 2 or additionally for alcohol consumption, red meat intake, fast food consumption, vegetable and fruit intake, and physical activity in model 3. This suggests that the association observed in the univariate analysis is influenced by these confounding factors.

Similar to our results, the prospective data collected by Poszler A. et al. from two centers in Germany show that after accounting for possible confounding factors (gender, age, diabetes, BMI, alcohol or nicotine abuse, indication for colonoscopy, withdrawal time), colonic diverticulosis was no longer significantly associated with the detection of adenomas [22].

As the study rightly points out, the study data concerning patients of Asian descent should be critically examined, such as the data from Hong W et al., where no statistically significant association between diverticulosis and adenomas or advanced adenomas could be established. This is because this study population showed significantly fewer cases of left-sided diverticulosis, which is more common in Western populations [13].

A comprehensive meta-analysis by Lee HJ et al., which included 29 cross-sectional studies and 450,953 patients, also showed no association between the occurrence of diverticulosis and advanced adenomas. However, regarding the overall rate of adenomas, there was an increase observed in patients with diverticulosis who exhibited gastrointestinal symptoms and did not undergo colonoscopy purely for CRC (colorectal cancer) screening purposes. These results suggest that the indication of colonoscopy could influence the rate of adenoma detection. Additionally, the described association was particularly evident in published studies, whereas unpublished data showed no such correlation [12]. Even though measures to avoid publication bias were taken by the authors, this must be considered overall. It should also be noted that there was no valid information available on the quality of adenoma detection or colonoscopy, and there are limitations regarding relevant confounding factors and their statistical correction.

A meta-analysis of 14 observational studies by Jaruvongvanich V et al. yielded contradictory results, showing that diverticulosis was associated with an increased risk of colorectal adenomas, but not with advanced colorectal adenomas and colorectal cancer [15]. Due to the statistical heterogeneity in different study designs, a lack of subgroup analysis and correction for confounding factors, as well as suspected publication bias, the association between diverticulosis and colon adenomas should be interpreted with caution.

It is strikingly evident that in studies with a positive association, such as those by Valvano M et al. [23] and Viscido A et al. [24], the indications of colonoscopy significantly differ from those without a statistically significant correlation, as previously mentioned. In the study by Valvano M et al., patients with a positive Hemoccult test, family history, or previous polypectomy were examined [23]. In the study by Viscido A et al., the examination was conducted due to lower abdominal pain or discomfort, hematochezia, changes in bowel habits, weight loss, iron deficiency anemia, chronic constipation, chronic diarrhea, and follow-up after colon polypectomy [24].

This reinforces the theory that the indication of a colonoscopy influences the adenoma detection rate, and therefore the hypothesized association should be critically examined. The data presented by Rondagh EJA et al., which describe a statistically significant correlation between adenomas, advanced adenomas, and diverticulosis in patients under 60 years old, are also based on a mixed patient cohort consisting of symptomatic patients, surveillance, and screening colonoscopies [14].

According to our results, there is currently no established common pathophysiological mechanism known to be responsible for the formation of both adenomas and diverticula. While dysplastic processes at the mucosal level are significantly responsible for the formation of adenomas [25], structural changes affecting the connective tissue, smooth muscle [26], and blood vessels [27,28] of the intestinal wall are likely associated with the formation of diverticula. Conversely, chronic inflammatory processes of the mucosa, which can also be detected microscopically in asymptomatic diverticulosis patients between the formed diverticula, could potentially promote the formation of adenomas [29].

Strengths of this analysis include its large number of participants and the comprehensive adjustment for potential confounding factors. However, there are also limitations concerning the study. The cross-sectional approach of our study captures the variables of interest at a single moment, offering only a limited view of the situation at that particular time. As a result, causal links cannot be established and shifts or trends over time cannot be evaluated. Because of the cross-sectional design, there is a potential for confounding variables that may not be entirely accounted for or controlled. Furthermore, in the asymptomatic screening population studied, the presence of selection and information biases, potentially influenced by factors such as general health awareness and public health outreach efforts, could affect the robustness of the findings. These biases may lead to an overrepresentation of individuals who are more health-conscious or have greater access to healthcare resources, thereby limiting the generalizability of the results to the broader population. Selecting a single center as the study location restricts the ability to generalize our findings to other geographic regions or population groups. Regional factors and patient profiles might be unique to the selected center and therefore may not accurately reflect the broader population of Western society. Additionally, the risk of a type II error cannot be excluded, meaning that some associations may have been missed due to insufficient power to detect smaller effect sizes. Because our study does not cover fundamental scientific elements, a detailed investigation at the cellular or molecular level is lacking.

Future research should focus on prospective studies that illuminate the causal relationships and underlying mechanisms between diverticulosis and colorectal neoplasms. This includes conducting long-term studies to assess the progression of diverticulosis and its potential impact on the development of colorectal cancer over time, clarifying whether changes in diverticulosis status correlate with shifts in neoplastic findings. Additionally, investigating specific biomarkers that may indicate inflammatory or neoplastic processes in patients with diverticulosis could help identify individuals at higher risk of neoplasia. Furthermore, further basic research at the cellular or molecular level is essential for a deeper understanding of these conditions.

## 5. Conclusions

In summary, our study revealed that patients with diverticulosis exhibit demographic and clinical features commonly associated with colorectal neoplasia, such as older age, male gender, and a higher prevalence of cardiometabolic risk factors. Importantly, the occurrence of adenomas in patients with diverticulosis appears to be incidental and bears no direct relationship to diverticulosis, as adenomas are common in the general population. While diverticulosis shares risk factors with colorectal neoplasia, our findings suggest that diverticulosis itself does not independently increase the risk of advanced colorectal neoplasia or unspecified polypoid lesions.

## Figures and Tables

**Table 1:** Baseline characteristics of patients with and without diverticulosis. (BMI: Body Mass Index; HGB: Hemoglobin; MCV: Mean Corpuscular Volume; CRP: C-reactive protein).

	No Diverticulosis	Diverticulosis	*p*-Value
	N = 3877	N = 2277	
AGE	56 (51–63)	62 (55–69)	<0.001
Age < 45 years	8% (298)	2% (39)	
Age 45–54 years	38% (1457)	20% (465)	
Age 55–64 years	34% (1321)	36% (824)	
Age 65–74 years	17% (642)	32% (723)	
Age ≥ 75 years	4% (159)	10% (226)	
SEX			<0.001
Female	50% (1930)	44% (1009)	
Male	50% (1947)	56% (1268)	
BMI	26 (23–29)	28 (25–30)	<0.001
Underweight	1% (39)	0% (6)	
Normal weight	40% (1534)	26% (582)	
Pre-obesity	39% (1529)	45% (1021)	
Obesity	20% (775)	29% (668)	
Hypertension	52% (2035)	67% (1523)	<0.001
Diabetes	16% (607)	20% (454)	<0.001
HbA1c	5.5 (5.2–5.7)	5.6 (5.3–5.8)	<0.001
Metabolic syndrome	73% (2836)	84% (1906)	<0.001
Creatinine	0.9 (0.8–1.0)	0.9 (0.8–1.0)	0.06
HGB	14.6 (13.7–15.4)	14.7 (13.9–15.5)	<0.001
MCV	87 (85–90)	87 (84–90)	0.38
Thrombocytes	232 (200–271)	226 (193–264)	<0.001
Leukocytes	5.7 (4.9–6.7)	5.9 (5.0–7.2)	<0.001
CRP	0.2 (0.1–0.3)	0.2 (0.1–0.4)	<0.001
ADENOMA			<0.001
No Adenoma	71% (2738)	63% (1438)	
One adenoma	18% (693)	22% (494)	
Two adenomas	6% (244)	8% (192)	
Three adenomas	3% (117)	4% (88)	
Four adenomas	1% (40)	1% (33)	
Five adenomas	1% (20)	1% (18)	
Six adenomas	0% (11)	0% (4)	

**Table 2:** Sensitivity analyses for sex, age, obesity (BMI > 30), and metabolic syndrome of advanced neoplasia and diverticulosis.

	Advanced Adenoma		
Subgroup	OR	CI 95%	*p*-Value
SEX			
Male	0.982	0.781–1.236	0.881
Female	1.331	0.956–1.851	0.09
METABOLIC SYNDROME			
Yes	1.078	0.88–1.322	0.465
No	1.068	0.644–1.772	0.798
AGE			
≤55 a	1.159	0.747–1.80	0.51
>55 a	0.897	0.726–1.108	0.313
BMI			
<30	1.227	0.984–1.529	0.069
≥30	0.845	0.59–1.21	0.357

**Table 3:** Sensitivity analyses for sex, age, obesity (BMI > 30), and metabolic syndrome of any polypoid lesion and diverticulosis.

	Any Polypoid Lesion		
Subgroup	OR	CI 95%	*p*-Value
SEX			
Male	1.284	1.111–1.483	0.001
Female	1.451	1.223–1.723	<0.001
METABOLIC SYNDROME			
Yes	1.260	1.116–1.422	<0.001
No	1.652	1.264–2.158	<0.001
AGE			
≤55 a	1.294	1.038–1.615	0.022
>55 a	1.141	1.000–1.301	0.05
BMI			
<30	1.401	1.232–1.593	<0.001
≥30	1.255	1.013–1.554	0.037

## Data Availability

The data presented in this study are available on request from the corresponding author due to ethical considerations and privacy concerns.
